# Dropouts From Sublingual Immunotherapy and the Transition to Subcutaneous Immunotherapy in House Dust Mite-Sensitized Allergic Rhinitis Patients

**DOI:** 10.3389/falgy.2021.810133

**Published:** 2022-01-05

**Authors:** Huan Chen, Guo-qing Gong, Mei Ding, Xiang Dong, Yuan-li Sun, Lang Wan, Ya-dong Gao

**Affiliations:** ^1^Department of Otolaryngology and Allergology, Central Hospital of Huangshi, Huangshi, China; ^2^Department of Allergology, Zhongnan Hospital of Wuhan University, Wuhan, China; ^3^Hubei Province Key Laboratory of Allergy and Immunology, Wuhan University, Wuhan, China

**Keywords:** transition, allergic rhinitis, allergic asthma, allergen immunotherapy, dropout

## Abstract

**Purpose:** Both subcutaneous immunotherapy (SCIT) and sublingual immunotherapy (SLIT) are effective in reducing symptoms and medication scores and inducing long-term efficacy in patients with allergic rhinitis (AR). However, SLIT has been associated with poor patient adherence. This study investigates the factors impacting dropout rates from SLIT in house dust mite (HDM)-sensitized AR patients.

**Methods:** A retrospective study was performed to analyze dropout rates and reasons in AR patients receiving *Dermatophagoides farinae* (*Der f*) SLIT with a follow-up period of 2 years.

**Results:** A total of 719 HDM-sensitized AR patients received *Der f*-SLIT. Dropout rates increased with time and most occurred after 1 year of SLIT. By month 24, 654 (91%) patients had discontinued SLIT. The dropout rates by month 24 were 100, 90.1, and 91.1% in children <5 years old, children aged 5–18 years old, and adults ≥ 18 years old, respectively. Combination with allergic asthma and mono- or multi-sensitization to other aeroallergens did not affect the dropout rates. The most common self-reported reasons for dropouts were refusal of continuation, dissatisfaction with the efficacy, transition to SCIT, and adverse effects. Refusal of continuation increased with age, whereas transition to SCIT decreased with age. Ninety-seven cases transitioned from SLIT to SCIT, and the transition rates increased with time. Comorbid allergic asthma did not affect the transition rates. However, multi-sensitization was associated with a slightly higher rate of transition to SCIT. The most common reason for the transition was dissatisfaction with the efficacy (54.6%), which was only reported by patients older than 5 years. For children who began SLIT at younger than 5 years old, the most common reason (81.2%) for transition was age reaching 5 years.

**Conclusions:** HDM-SLIT has a very high dropout rate, which is mainly due to refusal of continuation and dissatisfaction with the efficacy. Transitioning from SLIT to SCIT may help keep these patients on AIT and thus increase adherence and long-term efficacy.

## Introduction

The prevalence of both allergic rhinitis (AR) and allergic asthma (AA) have increased globally (1, 2), including in China (3–5), over the last 40 years. AR and AA have now become the most common respiratory diseases in children (6). Pharmacotherapy with inhaled corticosteroids is the mainstay of treatment for AR and AA, with the aims of ameliorating allergic inflammation, controlling symptoms, and improving lung function (6). Allergen-specific immunotherapy (AIT) is an etiological therapy aiming to induce immune tolerance to the culprit allergens, and offers the possibility of inducing specific tolerance beyond the duration of treatment and preventing the development of new allergic conditions (6). AIT has been proven to be effective in both seasonal and perennial AR when using seasonal pollen allergens or perennial allergen preparations, respectively.

Currently, two administration routes are available for AIT in both AR and AA: subcutaneous immunotherapy (SCIT) and sublingual immunotherapy (SLIT). SCIT was first performed approximately 110 years ago and is now widely accepted as a disease-modifying treatment for both AR and AA (6). AIT is used to prevent the progression of symptoms, the appearance of new sensitization, and the development of AA (7). Despite its efficacy, non-adherence to SCIT has been observed because of local and systemic adverse reactions, including anaphylaxis, and the inconvenience of repeated injections. SLIT is an alternative route for AIT that introduces allergen extracts to oral mucosal surfaces. SLIT can be administered by patients themselves at home (6). A few meta-analyses and systematic reviews have suggested the efficacy and safety of SLIT in both AR and AA (8–10).

House dust mite (HDM) allergens are the most common indoor aeroallergens causing AR and AA in southern China (11). HDM species *Dermatophagoides pteronyssinnus* (*Der p*) and *Dermatophagoides farina*e (*Der f*) are widely distributed in central and southern China (12). For AR and AA patients sensitized to HDM, both SCIT and SLIT with HDM drops or tablets are effective in reducing symptoms and medication scores, and in modulating immune responses (9). Most guidelines recommend only performing HDM-AIT in patients older than 5 years (6). There are limited data regarding the safety and efficacy of AIT in preschool-aged children (13).

HDM-SCIT is recommended as an add-on treatment for children and adults with controlled HDM-driven AA. HDM-SCIT is also recommended as an add-on treatment for adult AA patients to reduce allergen-specific airway hyperreactivity and improve quality of life (9). For children with controlled HDM-driven AA, SLIT with HDM drops is recommended as an add-on treatment to reduce symptoms and medication scores (9).

HDM-SCIT is not routinely administered in 3- to 5-year-old children, although a recent study found SCIT was safe in preschool-aged children (13). Children in this age group are unlikely to be able to perceive and promptly express if an adverse reaction occurs after the injection (6). SLIT with HDM drops had a similar efficacy and safety profile in children with AR aged 3–5 years and 6–13 years (14). Thus, HDM-SLIT offers a potential means to modify the natural course of atopic diseases for children aged 3–5 years with AR and with or without AA, because of its relatively better safety profile and higher efficacy when started at an earlier age (15).

Besides the allergens involved and route of administration, adherence to the treatment regime is another critical factor influencing the long-term efficacy of AIT. Although clinical and immunological changes occur during the early stages of AIT, international guidelines recommend a minimum of 3 years of treatment to achieve disease modification and long-term tolerance (16).

Previous studies have shown poor adherence and high dropout rates associated with SLIT. The dropout rates increase with the duration of treatment. The large proportion of dropouts has become a critical issue impacting the long-term efficacy of SLIT. However, previous studies have shown a greater effect of SCIT on clinical symptoms and immunological responses when compared with that of SLIT (17). While both SCIT and SLIT have greater efficacy than standard pharmacotherapy, SCIT is superior to SLIT in improving symptoms and medication scores of children with AR and AA in the first year of treatment (6). Moreover, greater immunological responses were observed with SCIT than with SLIT, in that higher allergen-specific IgG4 levels and decreased diameters of wheals in skin prick tests (SPTs) were only observed in SCIT patients. Other responses such as decreased reactions to a bronchial challenge and increased CD4^+^CD25^+^T cells were also observed only in SCIT patients (17). Therefore, some patients may need to change from SLIT to SCIT to achieve better clinical symptom relief and long-term efficacy.

In this study, we analyzed the proportions of AR patients in a tertiary medical center undergoing SLIT dropout and transition from HDM-SLIT to SCIT, in subgroups of patients with or without AA, as well as patients' reported reasons.

## Subjects and Methods

### Subjects

In this retrospective study, all patients diagnosed with AR combined with or without AA and receiving HDM-SLIT with the *Der f* drops “Chanllergen” in Huangshi Central Hospital from January 2019 to June 2021 were included. The diagnoses of AR and AA were made according to the Allergic Rhinitis and its Impact on Asthma (18) and GINA (Global Initiative of Asthma) guidelines (https://ginasthma.org/wp-content/uploads/2019/01/2018-GINA.pdf). Total serum IgE and allergen-specific IgE (sIgE) levels were assessed using standardized allergens from ImmunoCAP Phadiatop (Thermo Fisher Scientific, Sweden). Skin prick tests using the eight most common aeroallergens in Central China (19) were performed according to the guidelines of the European Academy of Allergy and Clinical Immunology (20). Wheal diameter was calculated as the mean diameter of the longest diameter and the diameter perpendicular to it. Tests with a mean wheal diameter ≥ 3 mm greater than the negative control were considered as positive. All patients received standard pharmacotherapy, including intranasal corticosteroids, antihistamines, inhaled corticosteroids, bronchodilators, and antileukotrienes. The study was approved by the Medical Ethics Committee of Huangshi Central Hospital (Approval Number: 20201-EBH-K004).

### Sublingual Immunotherapy

Confirmed consent was obtained from all patients or both parents of minor patients. The standard *Der f* extract drops “Chanllergen,” (Wolwo Bio-Pharmaceutics, Zhejiang, China), the first SLIT product approved by the China Food and Drug Administration in 2006 for the clinical treatment of AR and AA (21), were prescribed to patients sensitized to HDM and intended to receive SLIT with this product. The first dose was administered in the hospital under supervision. The patients or their parents were trained to use the drops at home. The drops were directly administered under the tongue and kept there for 1–3 min before swallowing, and no drinking was permitted for 15 min. The regimen for SLIT was as follows: Escalation phase: Vial 1–3 (1, 10, and 100 μg/ml of protein, respectively): 1, 2, 3, 4, 6, 8, and 10 drops for days 1–7 every week for Weeks 1–3; Maintenance phase: for children aged 3–14 years, Vial 4 (333 μg/ml of protein), three drops daily from Week 4; for those older than 14 years, Vial 4, three drops daily for Weeks 4–5, Vial 5 (1,000 μg/ml of protein), two drops daily for Week 6.

### Follow-Up of the Patients

The follow-up of the patients was performed by telephone calls in months 1, 3, 6, 12, 18, and 24 after the first treatment. During each follow-up call, patients were asked about their adherence to daily use, daily dose, symptom improvement, discomfort or any adverse reactions, and quality of life. The reasons for withdrawal from SLIT were also reported by the patients. For patients who transitioned to SCIT, their reasons were recorded at the first injection.

### Statistics

All data were analyzed with GraphPad Prism 8.0. Descriptive statistics were used to calculate the demographics, laboratory parameters, and percentages of dropout, and SCIT transitions for every visit. The Kruskal–Wallis test was used for continuous variables. The Chi-square test was used for categorical variables and the Fisher exact test for dichotomous variables. Tests with *P* < 0.05 were considered statistically significant.

## Results

### Baseline Demographic and Laboratory Characteristics

A total of 719 patients aged between 3 and 67 years (median age: 11 years) at the initiation of SLIT with *Der f* drops were included in this study. The demographics are summarized in Table 1. Forty patients were children aged 3–5 years, 477 patients were children aged 5–18 years, and 202 were adults. There were more male children aged 5–18 years. Five hundred eighty-six (81.5%) patients were diagnosed with AR only, while 133 (18.5%) patients had AR with AA. There were no significant differences in *Der p*-sIgE and *Der f*-sIgE serum levels between AR patients with and without AA (Supplementary Figures S1A,B). Three hundred eighty-eight (54.0%) patients had a family history of atopy, and there was no significant difference in the family history of atopy among the three age groups.

**Table 1:** Demographic characteristics and clinical signs at baseline.

	**Total (*n* = 719)**	**Children** **3–5 years** **(*n* = 40)**	**Children ≥5,** **and <18 years** **(*n* = 477)**	**Adults** **(≥18 years)** **(*n* = 202)**	***P-*** **value**
Age (years) median (range)	11 (3–67)	4 (3, 4)	9 (5–17)	32 (18–67)	–
Gender, *n* (%)					<0.001
Male	435 (60.5)	20 (50)	318 (66.7)	97 (48.0)	
Female	284 (39.5)	20 (50)	159 (33.3)	105 (52)	
Diagnosis, *n* (%)					
AR	586 (81.5)	36 (90)	381 (79.9)	169 (83.7)	0.185
AR+AA	133 (18.5)	4 (10)	96 (20.1)	33 (16.3)	–
Family history of atopy, *n* (%)	388 (54.0)	28 (70)	247 (51.8)	113 (55.9)	0.068
sIgE-Der p (KU_A_/ml), Mean ± SD	31.0 ± 31.3	43.5 ± 36.0^*^	29.9 ± 29.1	31.0 ± 31.2	0.024
sIgE-Der f (KU_A_/ml), Mean ± SD	29.2 ± 28.1	39.8 ± 34.2^*^	28.4 ± 27.4	28.9 ± 28.1	0.047
Other SPT^+^ allergens, *n* (%)	301 (41.9)	14 (35.0)	208 (43.6)	79 (39.1)	0.37
Cockroach	84 (11.7)	6 (15)	59 (12.4)	19 (9.4)	0.436
Alternaria alternata	71 (9.9)	3 (7.5)	48 (10.1)	20 (9.9)	0.873
Dog dander	38 (5.3)	3 (7.5)	24 (5.0)	11 (5.4)	0.815
Cat dander	59 (8.2)	1 (2.5)	41 (8.6)	17 (8.4)	0.399
Artemisia Pollen	68 (9.5)	3 (7.5)	52 (10.9)	13 (6.4)	0.174
Humulus scandens pollen	39 (5.4)	0	28 (5.9)	11 (5.4)	0.29

* *P < 0.05 compared with child 5–18 years and adults*.

All patients were sensitized to *Der p* and *Der f*, according to the skin prick tests and *Der f-* and *Der p*- sIgE results. Averaged *Der p*- sIgE and *Der f*- sIgE levels were higher in children aged 3–5 years when compared with those in children aged 5–18 years and adults. Three hundred and one (41.9%) patients also showed sensitization to other aeroallergens (multi-sensitized), such as cockroach, *Alternaria alternata*, dog and cat dander, *Artermisia* pollen, and *Humulus scandens* pollen, according to SPT. Serum *Der p*-sIgE levels were higher in multi-sensitized patients when compared with mono-sensitized patients. Serum levels of *Der f*-sIgE were similar between mono- and multi- sensitized patients (Supplementary Figures S1A,B). There was no significant difference in the percentages of the multi-sensitized cases among different age groups (Table 1).

### Dropout Rates in Different Age Groups

**Figure 1:**
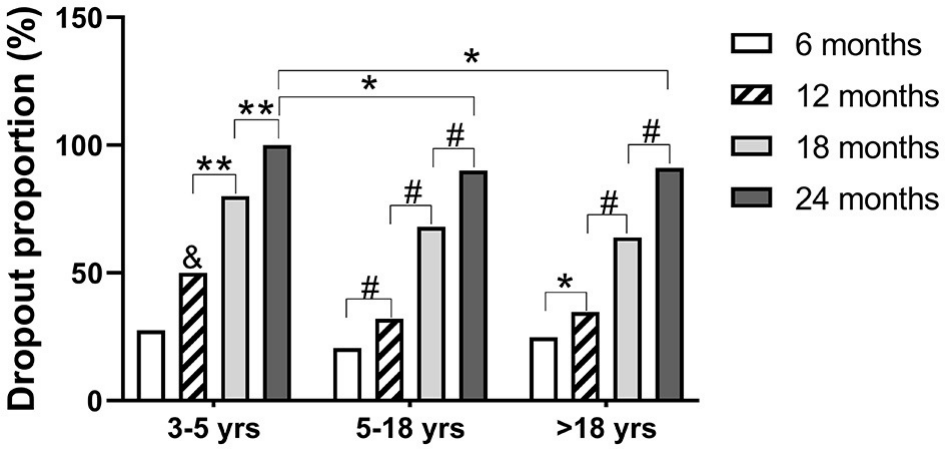
Dropout rates at different duration of SLIT. Patients were divided into three groups according to age when SLIT was initiated. The patients who transferred from SLIT to SCIT were also included in dropout cases. *P < 0.05; **P < 0.01; ^#^*P* < 0.001; & *P* < 0.05 compared with the dropout rate at 12 months of the 5–18 years age group. SCIT, subcutaneous immunotherapy; SCIT, sublingual immunotherapy.

In all three age groups, SLIT dropout rates increased significantly with time (Figure 1). The dropout rate in children aged 3–4 years increased to 100% by month 24 after initiation of SLIT, which was higher than in children aged 5–18 years (90.1%) and in adults (91.1%). Of note, the dropout rate in children aged 3–4 years was 50% by month 12 after initiation of SLIT, which is higher than in children aged 5–18 years (32.1%) and in adults (34.7%).

### Dropout Rates in AR With and Without AA and in Mono- and Multi-Sensitized Patients

**Figure 2:**
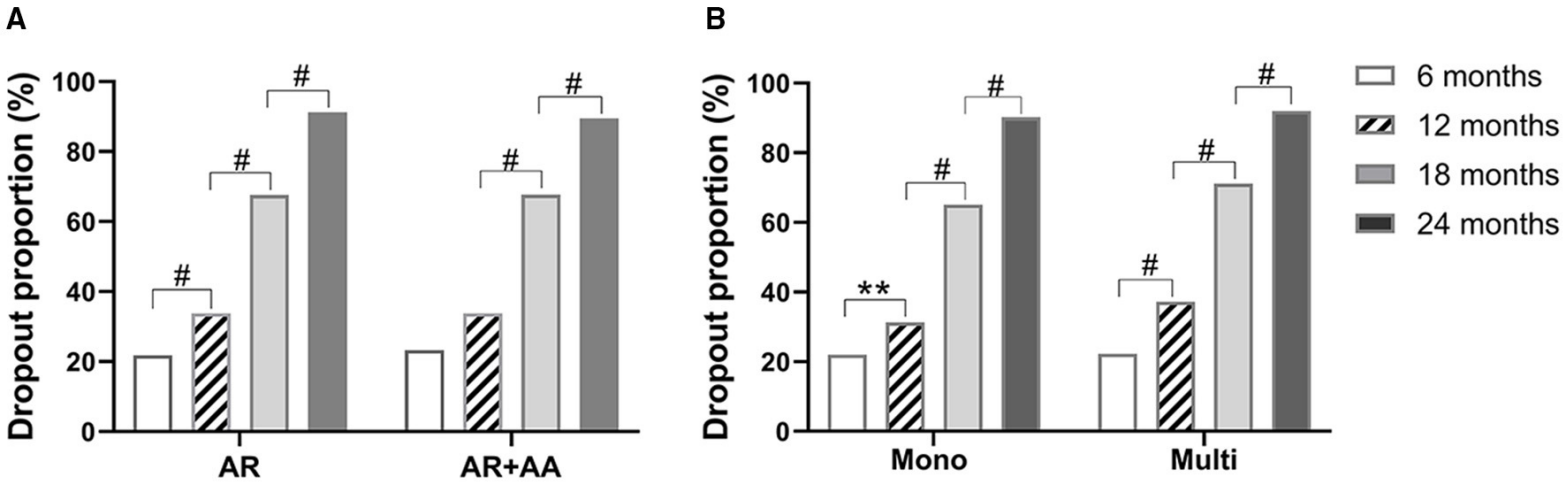
Dropout rates of SLIT in AR patients with or without AA **(A)**, and patients with different sensitization statuses **(B)** at different times. AR, allergic rhinitis; AA, allergic asthma. Mono, sensitized only to HDM; multi, sensitized to other aeroallergens in addition to HDM. **P < 0.01; ^#^*P* < 0.001.

The dropout rates were not significantly different between patients with AR alone and patients with AR and AA. The dropout rates increased with time in AR patients, whereas in AR patients with AA, most dropouts occurred during months 13–18. The dropout rates for AR patients with and without AA were 91.3 and 89.5% by month 24, respectively (Figure 2). These results imply that having AA as comorbidity does not impact the SLIT dropout rate in AR patients.

At all four time points, the SLIT dropout rates in HDM mono-sensitized patients were close to those in patients with multiple aeroallergens sensitization. The dropout rates by month 24 after initiation of SLIT were 90.2 and 92.0% in mono- and multi-sensitized patients, respectively. Similarly, the dropout rates increased significantly with time in both mono- and multi-sensitization patients (Figure 2). These results suggest that sensitization status does not impact the dropout rate of patients from SLIT.

### Reasons for SLIT Dropouts

**Figure 3:**
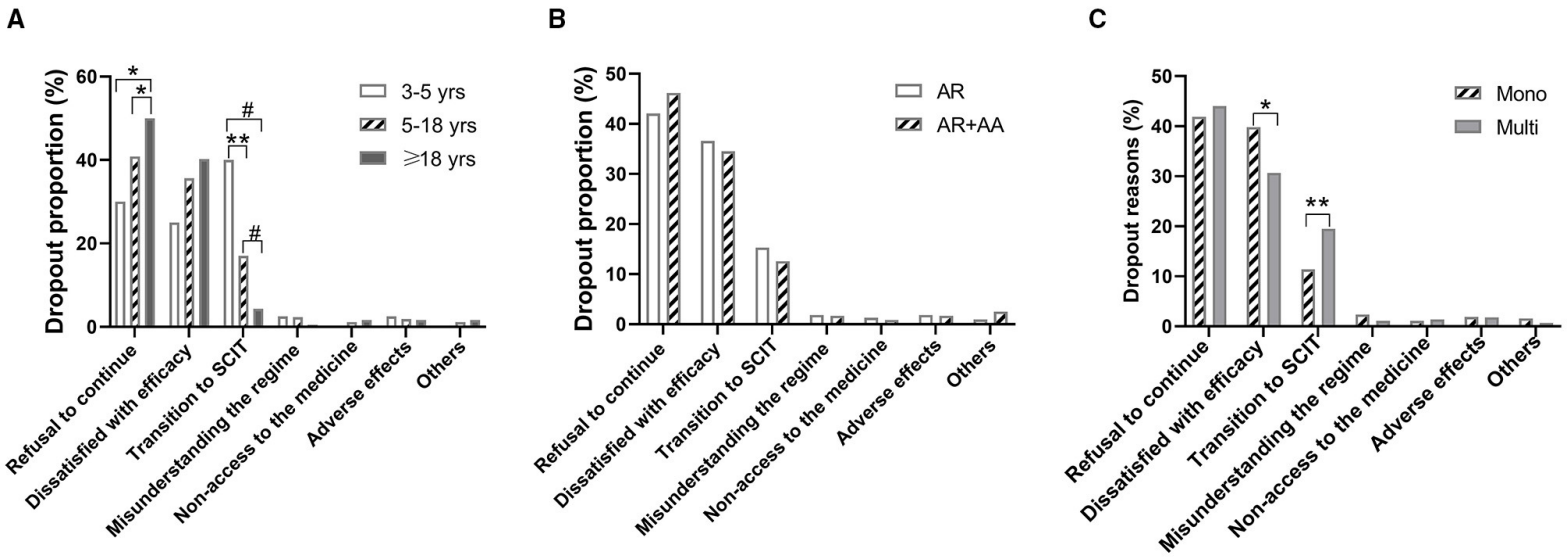
Dropout reasons according to age, underlying diseases, and sensitization status. **(A)** Dropout reasons in different age groups. **(B)** Dropout reasons in allergic rhinitis (AR) patients with and without allergic asthma (AA). **(C)** Dropout reasons in patients sensitized to HDM alone (Mono) and patients sensitized to other aeroallergens in addition to HDM (Multi). *P < 0.05; **P < 0.01; ^#^*P* < 0.001.

The major reasons for dropouts, as self-reported by patients or their parents, were refusal of continuation (42.8%), dissatisfaction with the efficacy (36.2%), and transition to SCIT (14.8%). Other reported reasons included also lacking access to the medicine (1.2%) and misunderstanding the SLIT regime (1.8%). Adverse reactions accounted for only eight (1.82%) dropouts (Table 2). Dropouts because of a refusal of continuation increased significantly with age, whereas dropouts due to transition to SCIT decreased with age (Figure 3A). Dissatisfaction with the efficacy as a reason for dropout had a trend of increasing with age, although without statistical significance (Figure 3A). The reasons for dropouts were not significantly different between AR patients with and without AA (Figure 3B). Interestingly, HDM mono-sensitized patients were more likely to withdraw from SLIT because of dissatisfaction with the efficacy, whereas multi-sensitized patients were more likely to discontinue SLIT and change to SCIT (Figure 3C).

**Table 2:** Reasons for dropout from SLIT or transition from SLIT to SCIT.

**Reasons**	**Dropout from** **SLIT, *n* (%)**	**Transition to** **SCIT, *n* (%)**
Refusal to continue	280 (42.8)	21 (21.6)
Dissatisfaction with the efficacy	237 (36.2)	53 (54.6)
Transition to SCIT	97 (14.8)	–
Adverse effects	12 (1.83)	5 (5.2)
Non-access to the medicine	8 (1.22)	–
Misunderstanding the regime	12 (1.83)	–
Reach 5 years of age	–	13 (13.4)
Other reasons	8 (1.22)	5 (5.2)
Total, 719	654	97

### Transition Rate of SLIT to SCIT in Different Age Groups

**Figure 4:**
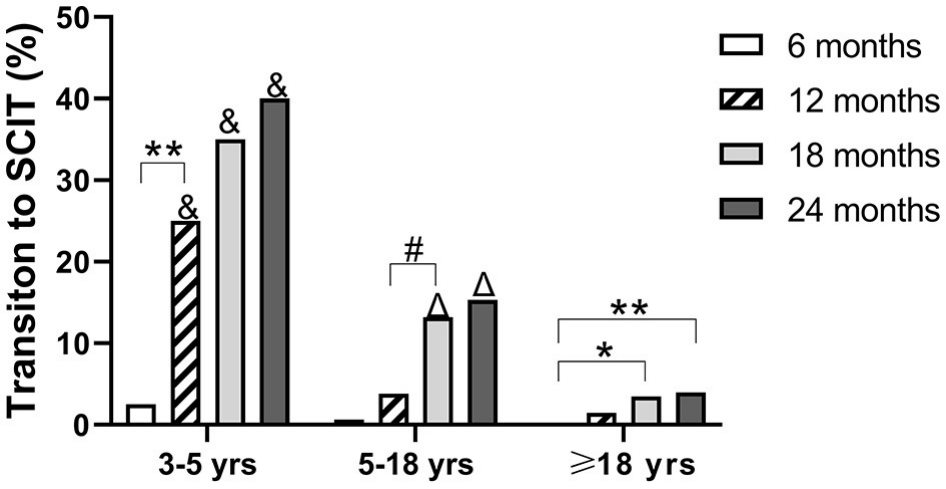
Proportion of patients changing from SLIT to SCIT at different times. Patients were divided into three age groups. *P < 0.05; **P < 0.01; ^#^*P* < 0.001; ^&^*P* < 0.001 compared with the transition rates of children at 5–18 and ≥18 years (adult) age groups at the same duration of SLIT; ^Δ^*P* < 0.001, compared with the transition rates of adults ≥18 years at the same duration.

In 719 patients treated with SLIT, 97 (13.5%) patients changed to SCIT during the 24 months of treatment. The averaged serum *Der p*- and *Der f*- levels were both higher in those patients who changed to SCIT when compared with those in patients without transition to SCIT (Supplementary Figure S1C). In these 97 patients, 16 (16.5%) were 3–5 years old, 73 (75.3%) were 5–18 years old, and eight (8.2%) were adults. In children aged 3–5 years, most of the SLIT to SCIT transitions occurred during months 7–12 after initiation of SLIT. However, in children aged 5–18 years and adults, most of the SLIT to SCIT transitions occurred during months 12–18 after initiation of SLIT (Figure 4). The transition rates in months 12, 18, and 24 were higher in children aged 3–5 years when compared with those at the same time points in children aged 5–18 years and adults. Also, the transition rates in months 18 and 24 were higher in children aged 5–18 years when compared with those at the same time points as adults (Figure 4). These data indicate that the SCIT transition rates increase with the duration of treatment and decrease with the age of patients.

### Transition Rates in AR Patients With and Without AA and in Mono- and Multi-Sensitized Patients

**Figure 5:**
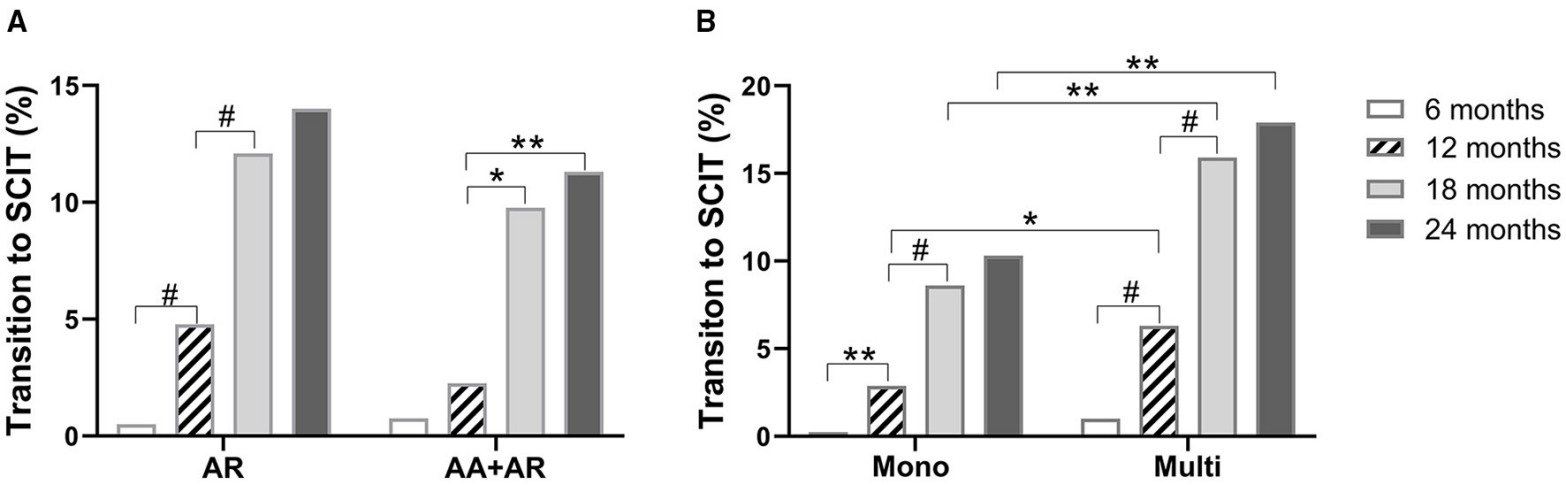
Proportions of SLIT to SCIT transition in AR patients with or without AA **(A)**. Patients with different sensitization statuses **(B)**. AR: allergic rhinitis; AA: allergic asthma. Mono: sensitized only to HDM; multi: sensitized to other aeroallergens in addition to HDM. *P < 0.05; **P < 0.01; ^#^*P* < 0.001.

Of the 97 patients who shifted from SLIT to SCIT, 82 (84.5%) were diagnosed with AR alone, while 15 (15.5%) were diagnosed with AR and AA. In patients with AR alone, most of the SLIT to SCIT transitions occurred during months 7–18, whereas in AR patients with AA, most of the SLIT to SCIT transitions occurred during months 12–18. At all these time points, the transition rates were not different between AR patients with and without AA (Figure 5A). These data suggest that having AA as a comorbidity does not impact the SLIT to SCIT transition rate.

In these 97 patients, 43 (44.3%) were solely sensitized to HDM and 54 (55.7%) were multi-sensitized. In both mono- and multi-sensitized patients, most of the transition from SLIT to SCIT occurred during months 7–18. Of note, multi-sensitization was associated with higher transition rates at months 12, 18, and 24 when compared with those of HDM mono-sensitized patients (Figure 5B). These results indicate that in patients sensitized to multiple aeroallergens, HDM-SLIT is associated with a higher probability of transition to SCIT.

### Reasons for SLIT to SCIT Transition

**Figure 6:**
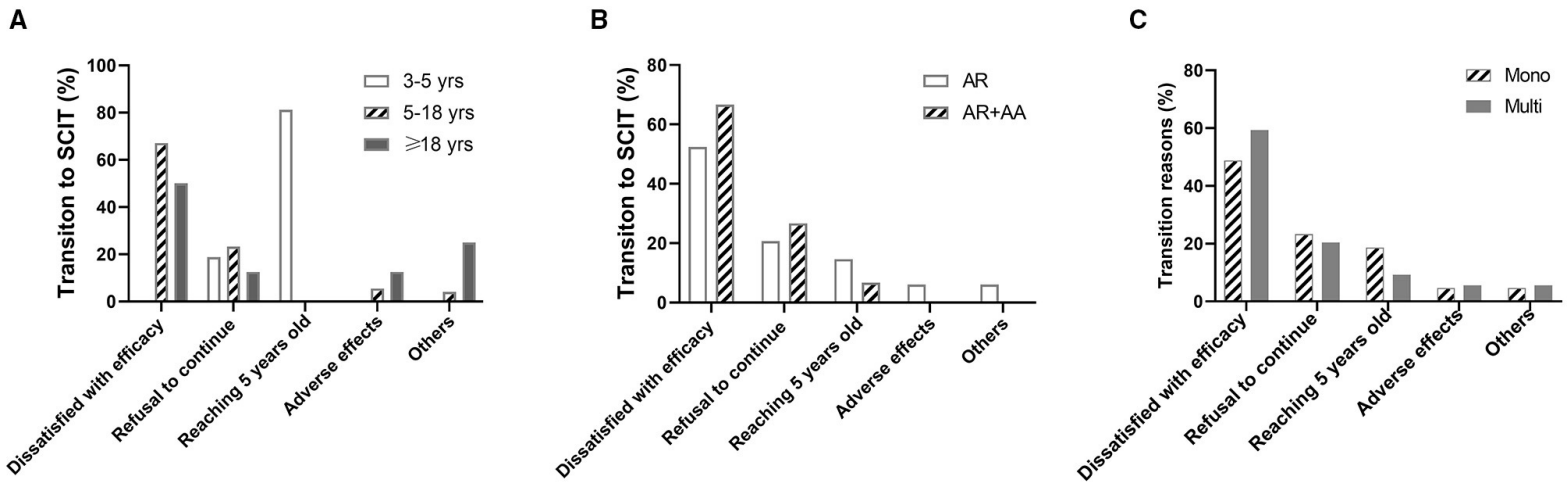
Reasons for transition to SCIT in patients with different ages, underlying diseases, and sensitization statuses. **(A)** Transition reasons in different age groups. **(B)** Transition reasons in allergic rhinitis (AR) patients with or without allergic asthma (AA). **(C)** Transition reasons in patients sensitized to HDM alone (Mono) and patients sensitized to other aeroallergens in addition to HDM (Multi).

In 97 patients who shifted from SLIT to SCIT, 53 (54.6%) reported the transition reason as being unsatisfied with the efficacy (inefficacy), 21 (21.6%) because they were unable to take the SLIT drops daily, 13 (13.4%) because they became older than 5 years, and only 5 (5.2%) cases because of adverse reactions to HDM-SLIT. Of note, in 16 children aged 3–5 years, 13 (81.3%) of the transitions were because they became older than 5 years. In 73 patients aged 5–18 years, the most common reason (67.1%) for the transition was dissatisfaction with the efficacy. In eight adult patients, 4 (50%) made the transition because of dissatisfaction with the efficacy (Figure 6A).

There was no difference in reasons for the transition from SLIT to SCIT between AR patients and AR with AA patients (Figure 6B). Dissatisfied with the efficacy was reported as a reason for the transition by more multi-sensitized patients than mono-sensitized patients, albeit without statistical significance. No significant differences were identified between the other reasons for the transition from SLIT to SCIT in mono-sensitization patients compared with multi-sensitization patients (Figure 6C).

## Discussion

The adherence to pharmacotherapy for the treatment of AR is very low (22). In this retrospective observational study, we found that SLIT had a dropout rate as high as 91% in AR patients during the 2 years of treatment. The dropout rate increased with time, and most dropouts occurred during the second year of treatment. An increasing dropout rate with the duration of SLIT was also reported by Kiel et al., who reported 62, 80, and 93% SLIT dropout rates at the end of the first, second, and third years, respectively. Market data from two manufacturers in Italy also found there was also a time-dependent increase in discontinuation of SLIT, with a dropout rate of 86.8% by the end of the third year (23). In contrast, a recent prospective study in China observed a much lower dropout rate of 36% at the end of 2 years of SLIT (24). Prospective controlled studies and double-blind, placebo-controlled randomized clinical trials have reported much lower SLIT dropout rates when compared with observational studies (25, 26). Dropout rates reported by other studies range from 23.2 to 65% (27–30). Interestingly, a study with a larger sample size showed a trend of a higher dropout rate compared with a study with a small sample size (25), which may also partly explain the high dropout rate in the current study. Of note, the adherence rate of SLIT is even lower than the dropout rate, when the number of days without medication is considered. These data suggest that adherence to SLIT has become a significant factor in the success and long-term efficacy of SLIT.

The efficacy of *Der f* drops product “Chanllergen” for SLIT has been proven by several studies (31–34), with a significant improvement in clinical symptom scores and immunological parameters such as the ratio of sIgE to sIgG4 (14, 35). This product has been recommended as a first-line treatment for AR in China by the Chinese Society of Allergy (21). Comparable efficacy and incidence of adverse events were observed between children aged 3–5 years and children aged 6–13 years (14) with the same *Der f* drops as in our study. However, the main reasons for dropout from SLIT, as self-reported by the patients, included dissatisfaction with the efficacy, nonadherence to daily use, transition to SCIT, and adverse reactions. Other studies reported that symptom improvement, financial issues, time constraints, changes of residence, and pregnancy were also reasons for dropout (28, 36). However, these factors were not documented in our study. In addition, administration of medicine at home and the lack of regular communication with a physician, who can offer support and encouragement to continue therapy (37), may also play a role in dropout from SLIT. Dissatisfied with the efficacy is a major reported reason for dropout from SLIT in the current and previous studies (24, 29, 38, 39). In younger children, refusal or subjective discomfort accounted for dropout in nearly half of the children, and dissatisfaction with the efficacy was rarely reported in younger children (40), which may be due to the lack of correct perception of the effects. In contrast, in older AR patients, loss of follow-up and relief of symptoms were the major reasons for dropout (41). Another study reported side effects as the major reason for dropout from SLIT (27).

Our study found that the underlying allergic disease, AR with or without AA, did not affect the SLIT dropout rates. A previous systemic review found a slightly higher dropout rate from SLIT in patients with AR and AA when compared with that in patients with AR alone, although without statistical significance (25). We found that the aeroallergen sensitization status, sensitized to multiple aeroallergens or solely sensitized to HDM, did not affect the dropout rates. This is similar to the results of a previous review (25).

Of note, the rate of transitions to SCIT also increased with time, and most of the transitions occurred 13–18 months after the initiation of SLIT. Younger children were more likely to change to SCIT when they reached the age of 5 years. AR patients were more likely to transition to SCIT when compared with those with AR and AA. Multi-sensitization was associated with a higher rate of transitions to SCIT. The main reasons for the transition were almost the same as the reasons for dropout.

Limited studies have focused on the transition from SLIT to SCIT (42). In our study, the transition rate from SLIT to SCIT was 13.5%, which is much higher than that reported by Leader et al. (1%). In contrast, they reported the transition rate of SCIT to SLIT was 16% (28). As expected, the transition rates increased with time, and most of these transitions occurred after 1 year of treatment, suggesting that 1 year is the expected period of efficacy for most patients. In our study, the transition rates were not significantly different between patients with AR alone and those with both AR and AA, even though AR patients with AA are more likely to be restricted to HDM-SCIT (9). In this study, the most common reason given for transitioning from SLIT to SCIT was dissatisfied with the efficacy, similar to that demonstrated in a previous study (42). However, only 5.2% of the transitions were due to adverse reactions, which is contrary to a study showing that most SCIT to SLIT transitions were due to adverse reactions to SCIT (42). Of note, in our study, among 40 children aged 3–5 years, 13 changed to SCIT after becoming older than 5 years. We speculate that the preferences of their parents and physicians toward SCIT may play a role in this transition.

We found that sensitization to multiple aeroallergens was associated with a significantly higher SCIT transition rate, but not dropout rate, at the end of the first year of SLIT compared with HDM mono-sensitization. This effect may be associated with a higher percentage of multi-sensitization patients feeling dissatisfied with the efficacy of SLIT when compared with that of mono-sensitization patients. In fact, some guidelines suggest that polysensitization to multiple allergens requires co-administration of effective and safe vaccines for each of these allergens (7).

Different approaches have been proposed to improve adherence to SLIT. Education, regular patient contact, and a follow-up plan could significantly decrease SLIT dropout rates (27, 43). Online platforms and digital tools such as short messages, emails, and social media may be used to increase adherence to SLIT. Allowing patients to choose the route of administration of AIT may also increase adherence to AIT (39). In our study, 13.5% of patients changed from SLIT to SCIT, and presenting this transition as an option could also be used to prevent patients from dropping out of AIT.

Our study had a few limitations. First, a portion of this study occurred during the Coronavirus Disease 2019 (COVID-19) pandemic in 2020, and the lockdown could have decreased access to the SLIT drops and impacted dropout rates and transitions to SCIT. Second, inefficacy was not judged based on an objective scoring of symptoms, and the self-reported motives for dropping out or transitioning may not reflect the true reasons. Finally, physician preference could not be ruled out as a potential reason for the transition in some cases.

In conclusion, the real-life dropout rates from SLIT increase with time, and dissatisfaction with the efficacy is the main reason for dropout. AR with and without AA and sensitization status are not the major factors influencing dropout. Certain patients may prefer to transition to SCIT and this could be a valid strategy to prevent these patients from dropping out of AIT. The major reasons given for transitioning from SLIT to SCIT are dissatisfaction with the efficacy and reaching the age of 5 years. For younger children with AR and AA, SLIT may be a transient treatment before initiating SCIT.

## Data Availability Statement

The original contributions presented in the study are included in the article/Supplementary Material, further inquiries can be directed to the corresponding author.

## Ethics Statement

The studies involving human participants were reviewed and approved by Medical Ethic Committee of Huangshi Central Hospital (Approval Number: 20201-EBH-K004). Written informed consent to participate in this study was provided by the participants' legal guardian/next of kin.

## Author Contributions

HC collected and analyzed the data. G-qG and LW contributed to data collection and patients follow-up. MD, XD, and Y-lS contributed to data analysis and manuscript writing. Y-dG designed the study, contributed to the data analysis, and writing. All authors contributed to the article and approved the submitted version.

## Conflict of Interest

The authors declare that the research was conducted in the absence of any commercial or financial relationships that could be construed as a potential conflict of interest.

## Publisher's Note

All claims expressed in this article are solely those of the authors and do not necessarily represent those of their affiliated organizations, or those of the publisher, the editors and the reviewers. Any product that may be evaluated in this article, or claim that may be made by its manufacturer, is not guaranteed or endorsed by the publisher.
