# Supplementary material for: Dropouts From Sublingual Immunotherapy and the Transition to Subcutaneous Immunotherapy in House Dust Mite-Sensitized Allergic Rhinitis Patients
Source: Front Allergy. 2022 Jan 5;2:810133. doi: 10.3389/falgy.2021.810133 (PMC8974757; doi:10.3389/falgy.2021.810133)
Supplement: Supplementary file 1 [file Data_Sheet_1.docx]

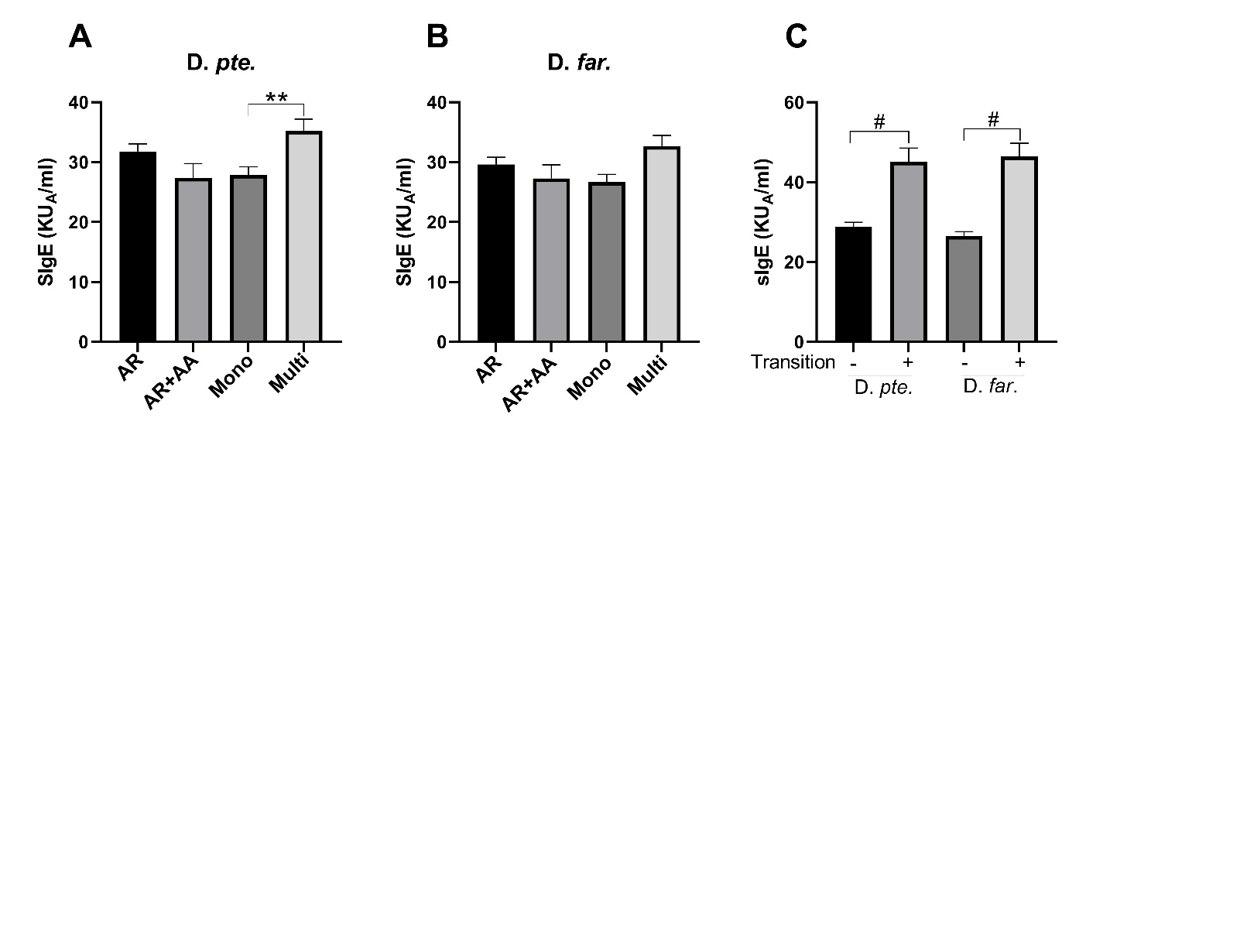


**Figure S1. Baseline serum HDM-specific IgE (sIgE) levels.**  **A, B.** serum D. *pteronyssinu*s-sIgE (**A**) and D*. farinae*-sIgE (**B**) levels in allergic rhinitis (AR) patients and AR patients with allergic asthma (AA), and in mono-sensitized and multi-sensitized patients. **C.** serum D. *pteronyssinu*s-sIgE and D*. farinae*-sIgE levels in patients with (+) and without (-) transition to SCIT. Shown are Mean ± SEM. **P < 0.01; #P < 0.001.
